# Towards a ‘Good Life’ for Farm Animals: Development of a Resource Tier Framework to Achieve Positive Welfare for Laying Hens 

**DOI:** 10.3390/ani3030584

**Published:** 2013-07-05

**Authors:** Joanne L. Edgar, Siobhan M. Mullan, Joy C. Pritchard, Una J. C. McFarlane, David C. J. Main

**Affiliations:** School of Veterinary Sciences, University of Bristol, Langford House, Langford, BS40 5DU, UK; E-Mails: Siobhan.mullan@bristol.ac.uk (S.M.M.); joy.pritchard@bristol.ac.uk (J.C.P.); um9614@bristol.ac.uk (U.J.C.M.); d.c.j.main@bristol.ac.uk (D.C.J.M.)

**Keywords:** animal welfare, certification, chicken, farm assurance scheme, good life, laying hen, positive welfare, quality of life, resource

## Abstract

**Simple Summary:**

Farm animals can be said to have a ‘good life’ if their quality of life is substantially higher than the current legal minimum and includes positive experiences such as pleasure. In commercial farms, animals can be provided with different resources such as bedding, exercise areas and enrichment objects. We used scientific evidence and expert opinion to determine which resources laying hens need to contribute to a ‘good life’. These resources were organised into three tiers, of increasing welfare, leading towards a ‘good life’. We describe how we developed the resource tiers and suggest how the overall framework might be used to promote a ‘good life’ for farm animals.

**Abstract:**

The concept of a ‘good life’ recognises the distinction that an animal’s quality of life is beyond that of a ‘life worth living’, representing a standard of welfare substantially higher than the legal minimum (FAWC, 2009). We propose that the opportunities required for a ‘good life’ could be used to structure resource tiers that lead to positive welfare and are compatible with higher welfare farm assurance schemes. Published evidence and expert opinion was used to define three tiers of resource provision (Welfare +, Welfare ++ and Welfare +++) above those stipulated in UK legislation and codes of practice, which should lead to positive welfare outcomes. In this paper we describe the principles underpinning the framework and the process of developing the resource tiers for laying hens. In doing so, we summarise expert opinion on resources required to achieve a ‘good life’ in laying hens and discuss the philosophical and practical challenges of developing the framework. We present the results of a pilot study to establish the validity, reliability and feasibility of the draft laying hen tiers on laying hen production systems. Finally, we propose a generic welfare assessment framework for farm animals and suggest directions for implementation, alongside outcome parameters, that can help define and promote a future ‘good life’ for farm animals.

## 1. Introduction

In accordance with the UK Farm Animal Welfare Committee’s (FAWC) Five Freedoms [1], farm assurance schemes and legislation have largely focused on the alleviation of negative aspects of welfare. Recently however, FAWC proposed that the minimum standards of farm animal welfare should move beyond the assessment of the Five Freedoms to achieve a ‘life worth living’ [2]. As an aspirational standard, in their 2009 report, FAWC also introduced the concept of a ‘good life’, representing the distinction that an animal’s quality of life is substantially higher than what implementation of legal minimum requirements can achieve, and over and beyond that of a ‘life worth living’ [2]. Indeed, it is now widely accepted that good welfare is not simply the absence of negative subjective states, but also includes the presence of positive experiences such as pleasure [3,4,5,6,7,8,9]. 

Within the scientific community there has been much discussion regarding suitable animal welfare definitions. Most definitions can be related to one or more of the following three broad ethical concepts: naturalness, physical state and mental state [10]. Dawkins [11] suggested that there is a “dangerous split that is now opening up between scientific definitions of animal welfare on the one hand and welfare as viewed by members of the general public on the other”. Scientists have focused on animal welfare outcomes that relate to health, physiology or behaviour, whereas consumers tend to value the naturalness concepts. However, Dawkins suggested that we should focus on two simple concepts; “We need to know both what the animals themselves want and what is good for their health.” Consideration of the health and an animal’s wants has, therefore been used to guide the framework below.

Overall on-farm assessment of animal welfare requires a combination of animal-based indicators to assess the actual state of welfare and resource-based indicators to identify risk factors [12,13,14,15,16]. Resource-based welfare assessment includes assessment of housing (e.g., floor/litter type), animal aspects (e.g., stocking density, breed and nutrition) and management practices. Animal-based welfare assessment is based on physical inspection of animals (e.g., feather loss, bone fractures), behavioural observations and farm records (e.g., disease incidence, mortality). Traditional animal-based measures focus on negative aspects of welfare, although one method, qualitative behavioural assessment (QBA)—an approach in which human observers summarise animals’ expressive demeanor—typically encompasses positive aspects of welfare, and has been incorporated into welfare assessment protocols, including Welfare Quality (e.g., for cattle [17]). Several organisations have recommended a more outcome-based approach to welfare assurance (FAWC [18], European Food Safety Authority [19], Farm Animal Welfare Forum [20] and OIE [21]). The practical application of this approach has been facilitated by the publication of standardised assessment protocols for numerous farm animal species (e.g., Welfare Quality [17]). 

As with negative aspects of welfare, the opportunity for animals to have positive experiences, which we describe as positive welfare, is probably best assessed on farms using a range of resource and animal-based measures to infer overall affective state as “there are as yet no feasible animal-based measures indicative of good welfare” [7]. FAWC [2] proposed that a ‘good life’ could be considered in terms of additional opportunities which was defined as a “resource that an animal does not need for biological fitness but is valued (*i.e.*, used) by the animal”. FAWC further proposed that the four opportunities: comfort, pleasure, interest and confidence, should be considered for the evaluation of the higher ‘good life’ standard. FAWC commented that “Provided that all other conditions were equal, then if an animal was to be provided with, and took, such opportunities, then it could be said to have had a better life.” Opportunities are therefore resources that are valued by the animals. FAWC are also clear that any harms arising from these resources should be minimised. For example, some animals value and consume energy dense food; however, an unrestricted access to this opportunity would be harmful in the longer term. Green and Mellor [22] discuss how the FAWC [2] concept of a ‘good life’ maps on to other quality of life assessments, for example the Five Freedoms [1] and critically evaluate its usefulness, citing the focus on positive welfare as its main strength. 

Other frameworks have also presented positive welfare in terms of broad concepts. For example, Mendl *et al.* [23] suggest an approach for understanding animal emotion using a two-dimensional system relating to ‘arousal’ and ‘valence’ giving rise to four types of affective state (low or high arousal coupled with positive or negative mood) with areas that discrete emotions can be mapped onto. They propose that positive welfare can therefore incorporate states such as ‘calm’ or ‘relaxed’, when coupled with low arousal levels, and ‘excited’ or ‘happy’ when more aroused. Mellor [24] discusses positive welfare in terms of ‘emotional action-orientated’ systems: ‘seeking’, ‘bonding’, ‘rage-assertiveness’, ‘care’, ‘play’ and ‘lust’ and suggests that incorporation of such concepts into codes of practice could serve to promote positive welfare in farm animals. Likewise, Yeates and Main [9] suggest positive welfare should be incorporated within policy instruments but propose that the assessment of positive outcomes be framed within the life aims animals have of “(A) everyday sensational pleasures, (B) engaging with their environment, their conspecifics and their handlers and (C) realising their own goals”. 

A more specific framework incorporating assessments of positive welfare was piloted by Mullan *et al.* [25] on 15 pig farms. Resource-based assessment criteria for five tiers of increasing welfare were defined for six elements of pig husbandry: environmental enrichment, foraging behaviour, thermal comfort, physical comfort, tail docking and floor space provision. Some outcome measures were found to correlate well with the welfare tier achieved for some elements, however, the large variation of outcomes achieved across farms within a welfare tier score suggested that a combination of resource and outcome assessments were required to provide an accurate assessment of positive pig welfare on farms.

To achieve the requirements of a resource- *and* animal-based approach to achieving positive welfare for farm animals, our aim was to apply scientific literature and expert opinion within an established thereotical framework, to determine which resources laying hens need to contribute to a ‘good life’. These resources would then be organised into three tiers, of increasing welfare, providing a reliable, practical resource tier framework, leading towards a ‘good life’. We aimed to use this iterative process to propose a common framework containing ‘good life’ opportunities and principles for farm animal welfare assessment. 

## 2. Materials and Methods

### 2.1. Underlying Principle

As discussed in the introduction, the resource tiers framework was based on the underlying principle and foundation that animals are considered to be in a good state of welfare if they ‘are healthy and have what they want’ [11]. This foundation necessitated the inclusion of resources that are both important to the species and compatible with the concept of providing individual animals with choice. 

### 2.2. Structure

Following identification of the general needs of the particular species (for example; comfortable physical environment and positive social interactions), these general needs could then be split according to FAWCs four opportunities; ‘Comfort’, ‘Pleasure’, ‘Confidence’ and ‘Interest’, based initially on the authors’, then later by the expert consultants’ judgment on what the needs achieved for the animal. FAWC’s four opportunities were considered as an authoritative framework, encompassing mental aspects of welfare. An additional opportunity; ‘Healthy Life’, was included to incorporate an achievable balance between animals being healthy and ‘having what they want’ [11]. Therefore, the framework consisted of distinct resources required for achieving a ‘good life’, categorised according to general needs, within the appropriate FAWC opportunity, set under the umbrella concept that the resources were compatible with providing individual animals with choice. 

### 2.3. Literature Review

At the beginning and throughout the resource tier construction process, we conducted an extensive review of literature relating to resources that would contribute to positive animal welfare, with the purpose of establishing new potential resources and to verify expert opinion. The literature search was conducted using Web of Knowledge. As well as reviewing papers comparing welfare outcome measures in different systems and with different resources, we utilised papers focusing on measuring preference for particular resources. 

### 2.4. Expert Consultation

As well as using published evidence, the resources required for a ‘good life’ were identified using the opinion of 12 experts from five academic institutions in the UK and New Zealand. The consulted experts were researchers who had extensive experience and knowledge of farm animal behaviour and welfare, judged by a relevant PhD qualification and their large number and broad range of relevant peer-reviewed publications and reputation within the field. The experts provided their views on which resources laying hens need to allow them to experience positive welfare. These experts also provided guidance on the relative ranking of resources, *i.e.*, which resources would be required to attain the three tiers of higher welfare; +, ++ and +++. The consultation was an informal, iterative process; as the draft resource tiers progressed, researchers were able to comment on and suggest amendments to the developing draft tiers. To manage disagreements, controversial opinions were put to subsequent experts, although no formal method of assessing agreement between the experts was employed. Throughout this process we cross-checked the draft resource tiers against the existing legislation and legislative codes of practice, to determine whether the resource tiers were (i) aiming for a consistently higher level of welfare and (ii) emphasising positive welfare and not simply absence of harm. As well as commenting on the scientific accuracy of the tiers, researchers with practical on-farm experience provided valuable feedback on whether the resources were practically achievable in a commercial setting.

### 2.5. External Review

After the construction of the tiers, these were presented in draft form to stakeholders from AssureWel; a collaborative project involving University of Bristol, Soil Association and RSPCA. These external stakeholders worked within the field of farm animal welfare assurance and had experience of laying hen production systems. Specifically, the stakeholders provided views on the relative ranking of the needs and resources, how practical the on-farm assessment might be, and provided insight into possible implementation. 

### 2.6. Pilot Study

Following construction of the draft laying hen resource tiers, a pilot study was undertaken to establish the validity, reliability and feasibility of the framework. Twelve farms, including organic (n = 1), free range (n = 3), barn (n = 3), caged (n = 2) and backyard (n = 3) systems were visited. Farm visits involved assessment according to the tiers and an interview with the producer. The assessment was carried out first, walking the sheds and range (if present) with the producer. Each farm was scored for their compliance with law and codes of practice and the criteria for Welfare +, ++ and +++ for each of the general needs. The assessor also rated how confident they were whilst assigning scores according to the framework using a 3-point scale: not very, somewhat or very confident. Any comments about the resource framework from both the assessor and producer were noted. Structured interviews with the producer ascertained their opinions on the draft framework. With the producers consent, these were recorded using a dictaphone. The producers were asked for their general opinions on the content of the framework and whether they thought the framework was relevant to their system. To help prompt discussion during the interview session, the producers were also asked to express their views on how useful the framework could be, how willing they would be to self assess, what they thought of the framework’s potential use in benchmarking against other farms or welfare schemes and the potential for adjustment of current scheme standards. After transcription the producer interview comments were ordered into key themes. 

**Table 1:** Laying hen resource tiers to assess compliance with legislation, code of practice and increasing levels of ‘good life’ opportunities (welfare +, ++ and +++).

**FAWC ‘Good life’ opportunity: *Comfort***		
**By choice of physical environment**	Law	Comfortable resting area. Access to well-maintained litter or a well-drained area for resting.
	Code	Floors, perches and platforms of suitable design and material to avoid discomfort, distress or injury to the birds. Perches of sufficient length to allow all birds to roost at the same time. Litter maintained in friable condition and at least 10cm deep.
***Birds should be able to exercise individual preferences for their physical comfort at all times***	Welfare +	As above, plus separate resting area and a choice of two or more types of suitable flooring (e.g., wood-based litter, peat substitute, straw, sand) **or** a choice of two or more types of perches (e.g., different diameters, shapes and materials).
	Welfare + +	As above, plus choice of two or more types of suitable flooring (e.g., wood-based litter, peat substitute, straw, sand) **and** a choice of two or more types of perches (e.g., different diameters, shapes and materials). Pullets should have had access to perch types and two or more suitable flooring types during rearing.
	Welfare + + +	As above, plus suitable flooring of a depth of >10 cm.
**By choice of thermal environment**	Law	Temperature kept within limits that are not harmful to the birds.
	Code	Housing provides shelter from adverse weather conditions or extremes of temperatures. Floors, perches and platforms kept sufficiently dry. Provision of insulation and ventilation to avoid heat and cold stress.
***Birds should be able to exercise individual preferences for their thermal comfort at all times***	Welfare +	**Choice** of temperatures **during the day** (e.g., gradient of suitable temperatures within the house). Protection from draughts in resting/perching area.
	Welfare + +	As above, plus a choice of temperatures **at all times**. Protection from weather on the range, if present (e.g., shade and windbreaks).
	Welfare + + +	As above, plus if range is provided, shelter from weather around access points (e.g., pophole roof, cover from wind and rain outside of popholes).
**By choice within environment while minimising harms**	Law	Protection from adverse weather conditions, predators and risks to the birds’ health. Accommodation and fittings for securing animals shall be constructed and maintained so that there are no sharp edges or protrusions likely to cause injury to them.
	Code	Nests, roosting areas, perches and platforms should not be so high above floor level that birds have difficulty using them or risk injury.
***Birds should be able to exercise preferences within their environment whilst minimising associated harms***	Welfare +	Perches positioned with safety in mind (e.g., above bird head height and below 1m above ground, adequate lighting around perches, no obstructions on the flight path below, angle between perches at different heights <45 degrees).
	Welfare + +	As above, plus measures for birds to safely traverse different levels (e.g., ramps between the litter and slatted area, if present) and safely accessible popholes, if provided).
	Welfare + + +	As above, plus policy for monitoring and acting on incidence of bone fractures.
**FAWC ‘Good life’ opportunity: *Pleasure***		
**By cognitive enrichment**	Law	At least 250cm^2^ of littered area per hen, litter occupying at least one third of the ground surface.
	Code	Littered area maintained in friable condition and at least 10cm deep.
***Birds should be able to experience positive emotional states through cognitive enrichment***	Welfare +	As above, plus daily access to complex structures to stimulate exploring or investigating (e.g., mazes, branches, **even** distribution of log piles, fallen down trees on the range), changed weekly.
	Welfare + +	As above, plus daily access to **more than one type** of complex structure to stimulate exploring or investigating (e.g., mazes, branches, **even** distribution of log piles, fallen down trees on the range), changed weekly.
	Welfare + + +	As above, plus daily access to learning enrichments (e.g., **even** distribution of feeding devices and tasks).
**By food choices**	Law	Fed a wholesome diet in sufficient quantity to maintain the birds in good health, satisfy nutritional needs and promote a positive state of wellbeing.
	Code	**In alternative systems**, wholegrain **may** be scattered over the litter each day. Regular access to insoluble grit to aid digestion.
***Birds should be able to exercise individual preferences for type of food and how it is obtained***	Welfare +	Complete diet **must** include an **even** distribution of wholegrain (e.g., wheat, barley, oats) and insoluble grit provided separately.
	Welfare + +	As above, presented in a way that interests the birds (e.g., scatterfed evenly or from a foraging device (e.g., pecking block). Feeders and drinkers on each level (e.g., litter and tiers).
	Welfare + + +	As above, plus an **even** distribution of forage crops (e.g., brassicas, grass, clover, peas, vetch, lupin, quinoa). Choice of feeder types (e.g., pan and chain feeders) and choice of heights of feeders and drinkers.
**FAWC ‘Good life’ opportunity: *Confidence***		
**By positive experience with stock keepers**	Law	Cared for by a sufficient number of staff who possess the appropriate ability, knowledge and professional competence.
	Code	Compassionate attitude. Where possible young birds should be given appropriate experience of management practices and environmental conditions. Frequent quiet but close contact with humans from an early age.
***Birds should be able to have positive experiences of stock keepers and husbandry***	Welfare +	Efforts to improve predictability/controllability for birds by signalling stressful events (e.g., knocking on the door before entering).
	Welfare + +	As above, plus birds experience different routines (e.g., different people and numbers of people, clothes, routes around house by stock keepers, playing the radio).
	Welfare + + +	As above, **from rearing period**, plus stock keepers regularly interact with birds, particularly in initial production period (e.g., talking to birds, maintaining regular visual contact, gentle touching, feeding from hand).
**By nesting choices**	Law	At least one nest for every seven hens. Where group nests are used, there must be at least 1m^2^ of nest space per 120 hens.
	Code	Nests with a floor substrate which encourages nesting behaviour.
***Birds should be able to exercise individual preferences for nest type and location***	Welfare +	Methods to minimise competition at the nesting area (e.g., at least one nestbox for every five hens. If group nestboxes are used, they must have partitions).
	Welfare + +	As above, plus methods to help birds to identify individual preferred nesting areas (e.g., several banks of nestboxes, different coloured or shaped nestboxes).
	Welfare + + +	As above, plus a **choice** of nesting floor substrates (e.g., wood shavings, buckwheat, oat husks) and depths of substrate.
**By positive social experiences within the flock**	Law	Several popholes at least 35 cm high and 40 cm wide, extending the entire length of building. 2 m per 1,000 hens. Feeding and watering equipment placed so as to **minimise** competition between birds.
	Code	All birds have sufficient access to feeding and watering equipment to **avoid** undue competition between birds.
***Birds should be able to have positive social experiences within the flock***	Welfare +	Resources are positioned to avoid competition between birds (e.g., food, water and enrichment spread out evenly). Policy for managing ‘pariah birds’ (e.g., by removing/culling).
	Welfare + +	As above, plus methods to create the perception of smaller group sizes (e.g., visual barriers such as bales of plastic-wrapped wood shavings, raised platforms.
	Welfare + + +	As above, plus enough space to allow birds to avoid negative social interactions. Smaller flock sizes. Cockerels if possible (housed separately).
**FAWC ‘Good life’ opportunity*: Interest***		
**By a positively enriched environment**	Law	At least 250 cm^2^ of littered area per hen, litter occupying at least one third of the ground surface.
	Code	Littered area maintained in friable condition and at least 10cm deep.
***Birds should be able to experience a rich environment throughout their lives***	Welfare +	An even distribution of **at least one type** of item to encourage foraging (breeze blocks, forage blocks, alfalfa blocks, chopped carrots, nets with cut straw/hay **and** manipulation (e.g., hanging items, CDs, stationary bunches of string/bailing twine, spherical objects) changed in form or presentation weekly.
	Welfare + +	An even distribution of **more than one type** of foraging and manipulation items as above, **from rearing period**.
	Welfare + + +	As above, plus extra measures to interest birds (e.g., projecting televised stimuli onto the walls, introducing novel objects at least weekly).
**By positive experiences of the outdoor environment**	Law	**If provided**, the range should be equipped with shelter from inclement weather and predators. Drinking troughs if necessary.
	Code	**If provided**, the range should provide reasonable precautions to protect against predators. Windbreaks on exposed areas of land. Provision of adequate, suitable, properly managed vegetation. Outdoor wholegrain feeding, a fresh supply of water, and overhead cover, all sufficiently far from the house to encourage the birds to range.
***Birds should be able to have positive experiences of the outdoor environment***	Welfare +	Measures to encourage confident and extensive use of the range (e.g., well-drained range with covered structures **and** hedges/shrubs, visible from the popholes and distributed evenly throughout the range, starting no further than 3m from the popholes. Drinkers on the range. Covered dustbathing opportunities (e.g., roofed sandpit) distributed evenly on the range, starting no further than 10m from the popholes. Other animals on the range (ruminants) if possible.
	Welfare + +	As above, before the onset of lay.
	Welfare + + +	As above, **from rearing period**. Substantial woodland/forest area for ranging.
**Additional opportunity: *Healthy life***		
**By dustbathing choices**	Law	**If provided**, open runs must be of an area appropriate to the stocking density and nature of the ground and equipped with shelter from inclement weather and predators and drinking troughs if necessary. Pophole access as per law for houses.
	Code	**If provided**, and included in the calculation of floor space, verandas must have the same artificial lighting system as the rest of the house. Pophole access as per law for houses and continuous access to veranda (if provided).
***Birds should be able to exercise individual preferences for dustbathing substrate and location***	Welfare +	**Continuous access** to a sheltered, naturally lit dustbathing area (e.g., veranda or shelter), with dustbathing substrate (e.g., wood-based litter, peat substitute, straw, sand) and adequate drinkers.
	Welfare + +	As above, plus measures to provide enough lighting in the dustbathing area during all seasons and weather conditions (e.g., daylight simulation bulbs during winter).
	Welfare + + +	As above, plus a **choice of** dustbathing substrates in the dustbathing area. Litter depth >10 cm.
**By effective management of day-to-day health and welfare**	Law	Animals which appear to be ill or injured shall be cared for appropriately without delay. Where they do not respond to such care, veterinary advice shall be obtained as soon as possible.
	Code	A health and welfare programme should be implemented for each unit which sets out health and husbandry activities. This should be developed with appropriate veterinary advice, reviewed against performance and updated accordingly. If the poultry are apparently not in good health, or showing obvious signs of behavioural alterations, the flock-keeper must take appropriate action without delay to establish the cause.
***Stock keepers should manage day-to-day laying hen health and welfare***	Welfare +	The health and welfare programme should be implemented and reviewed **frequently** plus action taken to **reduce or alleviate** the cause of any health and welfare problems. Routine use of medicines and mutilations should not be substitutes for good management.
	Welfare + +	As above, plus regular dialogue with veterinarian and scheme welfare advisor.
	Welfare + + +	As above, plus flock-keeper takes active part in welfare activities with wider benefits (e.g., member of scheme policy/ management group, peer advisor, on-farm welfare research).
**By positive genetic selection for long-term health and welfare**	Law	No animals shall be kept for farming purposes unless it can reasonably be expected, on the basis of their genotype or phenotype that they can be kept without detrimental effect on their health or welfare.
	Code	When considering the establishment or replacement of a flock, the choice of hybrid should be made with the aim of reducing the risk of welfare and health problems.
***Stock keepers should manage day-to-day laying hen health and welfare***	Welfare +	Farm manager recognises undesirable side-effects of genetic selection for production efficiency and chooses replacement animals to reduce/mitigate for current health and welfare problems within the flock (e.g., bone fractures, feather pecking).
	Welfare + +	As above, plus farm manager makes choices for potential **future** health and welfare issues within the flock, valuing these **equally** to egg production and other production factors.
	Welfare + + +	As above, plus farm manager chooses replacement animals for **long-term** improvement of flock health and welfare, resilience and metabolic normality, valuing these **over** egg production and other production factors.

## 3. Results and Discussion

### 3.1. Development of the Framework

The general needs as well as the resources required for a ‘good life’ were identified using both published evidence and the opinion of 12 experts from five academic institutions. During the literature search, we identified 121 scientific papers focusing on aspects of laying hen needs, resources or positive welfare. Consultation with experts resulted in distinct aspects of a ‘good life’, assigned to each of the five opportunities, and which were specific to laying hens. Table 1 presents the resulting draft resource tiers for laying hens. These tiers stipulate details of resources required to fulfill three tiers of welfare (welfare +, welfare ++ and welfare +++) according to the distinct aspects of a ‘good life’ under each of the five opportunities. To attain a ‘good life’ score of for any of the general needs, farms must also attain all of the lower scores, e.g., farms attaining a ‘good life’ score of +++, must also attain ++ and +, as well as adhering with the law and codes of practice relating to that particular need. As the umbrella concept was that the resources were compatible with providing animals with choice, the draft resource tiers stipulate the need for individual choice. The starting point was to attain the welfare +++ tier and work downwards. The law and codes are included for reference and refer to the current English law and codes of practice in 2013. 

The discussion with experts revealed six key themes, which were incorporated into the framework before the pilot study:

(1) *To achieve a ‘good life’, health and behavioural freedom should be delicately balanced.* Animals should not be provided with so much behavioural freedom that the impact on their health is greater than the behavioural benefit. For example, drinkers on the range may well encourage increased use by the birds, but drinkers have the potential to increase risk of disease transmission from wild birds. Hence, measures should be taken to preclude wild birds from using drinkers, along with the existing health plan. Conversely, a small impact on an animal’s health may be acceptable if this means that the animal has the choice to experience positive aspects of behavioural freedom that outweigh the harm.
(2) *Are the resource aspirational enough?* One expert suggested that we consider beginning from the natural situation—providing animals with a life as natural as they would experience in semi-wild or feral populations—and working downwards from this. However, this natural situation is immediately placed into jeopardy when we consider the fact farmed animals are reared for the sole purpose of food production. Other experts agreed that the resources should allow a more ‘natural life’ whilst maintaining that the resources *must* be compatible with production. For example, certain dairy systems allow cows to raise their own young to a certain extent. However, allowing a hen to raise her own chicks would be detrimental to production in both the hen and the chicks, causing the hen to cease laying for this period and limiting the number of chicks that could be raised.
(3) *Minimising harms is paramount to promoting positive welfare.* One expert stated that “minimising harms frees an animal up to enjoy the positives”. Whilst it was acknowledged that prevention of harm, allowing an animal to have a ‘life worth living’ [1], focuses on negative aspects of welfare, experts agreed that the absence of such harms is paramount to the generation of a positive welfare state. For example, the absence of thermal discomfort will likely allow an individual to divert time and energy away from thermoregulation and towards activities which contribute to positive aspects of welfare, e.g., positive interaction with enrichment or ranging behaviour. Indeed, the construction of the tiers resulted in these two distinct notional categories of needs; those needs that would prevent harm and those that would facilitate positive welfare.
(4) *Outcome-based welfare assessment should be incorporated into the framework.* It is important to link resources and outcomes within the framework, so that the aim of the tiers—positive welfare—can be assessed on an individual or group level. As the resource tiers progressed, it became evident that, whilst the main focus of the framework was resource-based measures of welfare, many of the opportunities would need to be assessed using outcome-based measures of welfare. For example, ‘enriching the environment’ could be assessed by recording the enrichments provided, or using the outcome measure of the number of birds interacting with enrichment, as well as estimating the contribution to a ‘good life’ through welfare indicators like changes in stress hormones or behavioural fearfulness scores.
(5) *The framework should provide scope for modification if future research dictates this*. During the literature review it became evident that, whilst numerous papers on resource use exist, there is a definite lack of empirical research into measures of, and resources required for, positive welfare in laying hens. Traditionally, research has focussed on indicators of negative states in animals, and although the presence of positive states has been highlighted as an area of future importance, there have been few studies into positive welfare indicators in animals. Experts agreed that there is a need for further research into positive welfare indicators and resource needs in laying hens. The framework should hence be a dynamic and up-to-date tool.
(6) *Producers should be encouraged to provide input into the tiers*. Examples of resources given within the framework should only be used as a guide and producers should be encouraged to engage in discussion with scheme representatives and the industry to pilot ideas for resources. These could be novel in type of presentation, so long as they facilitate expression of the general needs.

### 3.2. Pilot Study

It was possible to assign scores to all resource needs for all farms visited, using both inspection and interviews with producers. The time taken to complete the assessment for each farm was around 30 minutes for all systems. The numbers of farms attaining ‘good life’ scores are detailed in Table 2. Table 3 highlights the ease at which the assessor assigned compliance with each resource tier, presented as confidence scores (‘not very’, ‘somewhat’ and ‘very’ confident). The vast majority of the needs were scored either ‘very’ or ‘somewhat’ confidently. A potential problem was highlighted whilst scoring some systems for ‘breeding for positive welfare’. It was found that it was difficult to ascertain how motivated producers were to influence long term health and welfare, leading to a subjective evaluation. It was determined that a more objective approach might be to look at the records for breeding/breed choice and to evaluative how suited the breed is to the management system. 

It was noted by the assessor that, where some of the resource needs were assessed only ‘somewhat’ or ‘not very’ confidently, this was due to ambiguous interpretation of the resources descriptions. For example the ‘Confidence: Positive experience with stockpersons: Welfare +’ requirement stipulates that there should be ‘efforts to improve predictability/controllability for birds by signalling stressful events (e.g., knocking on the door before entering)’. To reduce the possibility of subjective interpretations, more examples could be provided. Additionally, adequate and regular training of assessors would ensure accurate scoring and agreement between assessors. 

The twelve farms visited included organic (n = 1), free range (n = 3), barn (n = 3), caged (n = 2) and backyard (n = 3) systems. Although the purpose of the pilot study was not to compare systems, it is clear that some elements of the resource tiers are not possible to provide in certain systems. The caged production system was attributed ‘good life’ scores (*i.e.*, scores above the codes and legislation) for only 3 of the 13 resource needs, compared to 9 for barn and all 13 for free-range. It is, therefore, expected that any application of this system to a larger number of farms will inevitably highlight differences between laying hen management systems. In order to promote welfare improvement within systems it is also hoped that the system will highlight a range of scores within systems. Even within this small study there was evidence that there was variation within systems. For example one free range farm attained ‘good life’ scores for only 9 of the 13 resource needs.

**Table 2:** The number of farms attaining compliance with criteria for ‘good life’ tiers, obtained during assessment using the ‘good life’ resource tiers (farms must attain ‘good life’ scores for welfare + to be considered for welfare ++ and +++).

Opportunity	Resource need	Number of farms attaining ‘good life’ scores		
		Welfare +	Welfare ++	Welfare +++
**Comfort**	**Comfortable physical environment**	5	3	2
	**Comfortable thermal environment**	12	7	5
	**Safe environment (opportunities to avoid physical hazards)**	8	7	2
**Pleasure**	**Enhanced learning opportunities**	7	7	6
	**Food enrichment**	9	3	3
**Confidence**	**Positive experience with stock keepers**	12	9	4
	**Facilitating egg laying**	7	4	2
	**Promoting positive social interactions**	11	5	5
**Interest**	**Enriching the environment**	6	4	3
	**Promoting ranging**	6	1	1
**Healthy life**	**Opportunities to dustbathe**	6	5	5
	**Management policy for positive health**	10	9	8
	**Breeding for positive welfare**	7	3	0

**Table 3:** Confidence scores in assigning ‘good life’ scores (number of farms), obtained during assessment using the ‘good life’ resource tiers.

Opportunity	Resource need	Number of farms attaining confidence scores								
		Welfare +			Welfare ++			Welfare +++		
		Not very	Somewhat	Very	Not very	Somewhat	Very	Not very	Somewhat	Very
**Comfort**	**Comfortable physical environment**	0	4	8	1	2	9	1	3	8
	**Comfortable thermal environment**	1	2	9	0	1	10	0	0	11
	**Safe environment**	0	5	7	0	2	10	2	5	5
**Pleasure**	**Enhanced learning opportunities**	0	0	12	0	0	12	6	2	4
	**Food enrichment**	0	1	11	1	3	8	1	2	9
**Confidence**	**Positive experience with stock keepers**	0	4	8	0	2	10	4	5	2
	**Facilitating egg laying**	0	2	10	0	0	12	0	1	11
	**Promoting positive social interactions**	3	8	1	0	3	9	0	3	9
**Interest**	**Enriching the environment**	0	2	10	1	3	7	0	1	11
	**Promoting ranging**	1	4	6	0	3	8	3	3	5
**Healthy life**	**Opportunities to dustbathe**	0	5	7	2	1	9	1	2	9
	**Management policy for positive health**	2	2	8	0	1	11	0	0	12
	**Breeding for positive welfare**	5	6	1	4	5	3	5	1	6

Producers were generally keen to discuss the framework and were often supportive of the concept. The following five common themes arose during discussion of the framework and its application.

(1) *Scientific validity of resource tiers.* Several producers expressed a general distrust of the science behind the framework. When talking about enrichment devices to stimulate exploration and investigation (Pleasure: By cognitive enrichment: Welfare ++) one farmer said “I would have to question whether those are actually a welfare contribution. It is from a human perspective but is it from a chicken perspective? I don’t know that and I’m sure you don’t know that and I'm sure a lot of other people don’t realise that”. One producer described the framework as “evolutionary (with potential to) lead in the right direction” but warned that “just making legislation without researching it properly is a bad thing”. Successful use of the framework would have to involve assurances for producers that the framework is backed by solid empirical evidence.
(2) *Relative value placed on health compared to behavioural choice.* Some producers felt that health is far more important than behavioural aspects of welfare, whereas the framework places health as an equally important aspect of overall welfare. Indeed, one producer identified a direct contradiction; “if you try to encourage a bird to look at different feeding devices, log piles, fallen down trees, that brings in a whole host of other different elements of an ecosystem, of bugs of bacteria of insects…..So giving them access to more areas which are potentially dirty potentially hold more problems to me. [This sounds]…cosmetically fantastic, but from a practical point of view is lunatic…. probably advocating more treatment and medication for birds”.
(3) *Reliability of assessment.* Interviews with producers revealed that some had concerns on the reliability of the assessment. Particularly, producers were concerned that environmental factors, beyond the producers’ control, might compromise their scores. For example one farmer stated that “A good farm suddenly turns into a bad farm because something has stressed the flock which was outside of the farmer’s control”. Another farmer added that “Weather is everything. Certain places out in the country don’t get the same weather as we do here.” It would therefore be recommended that assessment should be carried out during a variety of seasons to take account of weather and events that are beyond the producer’s control.
(4) *Ability to highlight a range of performance within systems.* Some producers highlighted the framework’s potential value to distinguish between ‘good’ and ‘bad’ farms within the same system. For example, one producer stated that “commercial free range probably varies vastly. There isn’t any way of grading how good that free range farm is *versus* another.” Another farmer agreed, introducing the term “intensive free range”, and questioning “do those birds range to the letter of the law? On the space allowance required? No. Some birds do but do they all roam? No. Of course they don’t.”
(5) *Costs of implementation.* With respect to possible implementation options some producers had reservations about any additional costs that might be incurred to achieve higher welfare tiers. For example, one farmer stated that “the more choices you give to the bird at the end of the day someone has to pay for them. And is anybody willing to pay for them?” There were also concerns about the time commitment associated with increased numbers of inspections.

### 3.3. Application to Other Species

Taking into consideration themes arising from engagement with experts, producers and the assessor, we propose a common framework for use in all farm animal species. Application to other species was considered important and a set of generic overarching opportunities and principles are presented in Table 4. The specific opportunities of ‘facilitating egg laying’, ‘opportunities to dustbathe’ and ‘promoting ranging’ were removed as they were considered to be sub-sets of other opportunities. For example, the positive welfare experienced through ‘promoting ranging’ could be assessed under ‘enriching the environment’. Additional opportunities, not previously discussed with experts or pilot tested were ‘promoting telos’, ‘play’ and ‘breeding and nurturing’. All were considered to afford specific opportunities for positive welfare not adequately captured elsewhere and it is recognised that some opportunities cover more than one aspect. For example, ‘play’ can promote positive welfare through pleasurable experiences and also confer life advantages for animals that have experienced play, particularly in early life [26]. Defining the tiers for each opportunity is in progress for pigs and cattle and further work could determine how applicable this framework is to other farmed species, and even other domestic and captive animals.

**Table 4:** Generic ‘good life’ opportunities and principles for farm animals.

‘Good Life’ Opportunity	Principle
**Comfort**	
Comfortable physical environment	Animals should be able to exercise individual preferences for their physical comfort at all times
Comfortable thermal environment	Animals should be able to exercise individual preferences for their thermal comfort at all times
Safe environment	Animals should be able to exercise preferences within their environment with minimum risk of harm
**Pleasure**	
Food enrichment	Animals should be able to exercise individual preferences for type of food and how it is obtained
Play	Animals should be able to exercise individual preferences for play
Breeding and nurturing	Animals should be able to have positive reproductive and nurturing experiences
**Confidence**	
Positive experiences with people	Animals should be able to have positive experiences of people when encountered
Promoting positive social interactions	Animals should be able to have positive social experiences within their group
**Interest**	
Enriched environment	Animals should be able to experience a rich environment throughout their lives
Enhanced learning opportunities	Animals should be able to experience positive emotional states through cognitive enrichment
**Healthy life**	
Management policy for positive health	Animal carers should manage day-to-day animal health effectively
Breeding for positive welfare	Animal carers should positively influence the long-term health and welfare of animals
Promoting telos	Animals should be able to live a life free from mutilations

## 4. Discussion

In this article we have further expanded the concept of a ‘good life’ as proposed by the Farm Animal Welfare Council [2]. Both the experts and producers identified the importance of scientific validity of the framework. Whilst we endeavoured to review the literature and consult scientists with a good understanding of the empirical research, there remains scope for differing opinions outside this field of experts. The development method was designed to address face (expert opinion) and content (encompass necessary domains) validity [27]. However, further work would be needed to evaluate the construct validity of this system by exploring the relationships between scores and other measures of welfare. Additionally, on-going empirical research into laying hen welfare, and technological developments in the industry mean that the framework should allow scope for change. Continued development and external scrutiny is therefore necessary to ensure an evidence-based and transparent approach. In focusing on positive rather than negative aspects of welfare, the proposed generic ‘good life’ opportunities and principles presented in Table 4 are not intended as a holistic framework. A holistic approach would also require inclusion of criteria that focused on harms. Other frameworks are available that encompass these negative aspects. For example at least four of FAWC’s five freedoms [1] are explicitly focused on negative aspects. Also, whilst the Welfare Quality principles are described in terms of good feeding, good housing, good health and appropriate behaviour, the detailed measures within the protocols for each species are mostly focused on negative parameters except for the qualitative behavioural assessment which is described as a criterion for positive emotional state [17]. 

As with other welfare assessment methodologies, this ‘good life’ framework could have application as a research, certification/legislation or management tool [13]. One potential application is to facilitate comparison between certification schemes. Worldwide, numerous food certification schemes have been developed to provide assurances to consumers on animal welfare and other societal concerns [28,29]. However, in their report of 2011 [17], the Farm Animal Welfare Committee (FAWC) suggested that there is considerable confusion in relation to food provenance. FAWC recommended that “independent governance is needed to align higher welfare claims to a common and identifiable set of defined welfare objectives and outcomes against which welfare claims can be compared directly by interested consumers”. Although the framework presented here could contribute towards a better understanding of schemes, it does not include an on-farm assessment of welfare outcomes, such as feather loss and injury. LayWel reviewed data sources of health and welfare outcomes allowing comparison of laying hen husbandry systems [30]. As explicitly recommended by FAWC and highlighted by experts involved in the consultation, it is important that any scheme comparison should also include information on welfare outcomes. Collation of information on outcomes has been instigated in some certification schemes [31]. Whilst this framework may be useful for examining scheme standards, it does not include an assessment of the implementation of standards. Scheme comparison should, therefore, also include an assessment of the credibility of the certification scheme assurance processes. 

There are a number of other possible applications for the framework proposed here. The framework could also be used by certification schemes to guide future standards development. For example, a scheme currently requiring welfare + in certain criteria may aim to change standards that ensured welfare ++ criteria were achieved within a defined timescale. By reporting the proportion of member farms that achieve each resource tier, the framework could also be used by schemes to claim even higher welfare standards amongst their members. This would require that all farms within a scheme were routinely assessed against this framework. Furthermore, if benchmarked data were provided to scheme members, then individual farmers might also use the data as a management tool. Some farmers might be motivated to change husbandry conditions to achieve higher levels on individual criteria. Additionally, the framework could be used by legislators as a policy tool to enable comparison of different schemes and method of production descriptors. If resource tiers were found to be related to compliance with welfare legislation, then the framework could perhaps also be used as a risk assessment measure influencing frequency of cross-compliance assessments. 

If the assessment were to be included within the surveillance procedures of certification schemes, then it would be important to justify the increased time taken in terms of welfare benefits or increased transparency of the scheme. The pilot evaluation described in this study showed that the assessment took approximately 30 minutes. Since it is also relatively uncomplicated, farmers could also undertake self-assessment. Although self-assessment might compromise the credibility of the assessment, a proportion of farmer responses could be verified during routine surveillance visits. Farmer involvement with the framework might also promote its interest as a management tool. Further practical evaluation, including assessment of consistency, would be needed before wider application. 

## 5. Conclusions

The ‘good life’ resource tiers framework proposed here could provide a contribution to the evaluation of positive welfare. This may be useful to promote higher standards of welfare for farm animals and for promoting transparency for consumers. The framework appears to be relatively easy to use during farm visits and has the potential to highlight differences both within and between different husbandry systems. Welfare scientists and producers consulted during its development, whilst often broadly supportive of the principles, raised important concerns that need to be considered in future development, such as scientific validity, avoidance of harm (as well as promotion of positive welfare) and concurrent assessment of health and other welfare outcomes. 

## References

[1] Brambell Committee (1965). Report of the Technical Committee to Enquire into the Welfare of Animals Kept under Intensive Livestock Husbandry Systems.

[2] (2009). Farm Animal Welfare in Great Britain: Past, Present and Future.

[3] Fraser D., Duncan I.J.H. (1998). Pleasures’, ‘Pains’ and Animal Welfare: Toward a Natural History of Affect. Anim. Welfare.

[4] Desire L., Boissy A., Veissier I. (2002). Emotions in Farm Animals: A New Approach to Animal Welfare in Applied Ethology. Behav. Process..

[5] Mendl M., Paul E.S. (2004). Consciousness, Emotion and Animal Welfare: Insights from Cognitive Science. Anim. Welfare.

[6] Burgdorf J., Panksepp J. (2006). The Neurobiology of Positive Emotions. Neurosci. Biobehav. Rev..

[7] Boissy A., Manteuffel G., Jensen M.B., Moe R.O., Spruijt B., Keeling L.J., Winckler C., Forkman B., Dimitrov I., Langbein J., Bakken M., Veissier I., Aubert A. (2007). Assessment of Positive Emotions in Animals to Improve Their Welfare. Physiol. Behav..

[8] Bracke M.B.M., Edwards S.A., Metz J.H.M., Noordhuizen J., Algers B. (2008). Synthesis of Semantic Modelling and Risk Analysis Methodology Applied to Animal Welfare. Animal.

[9] Yeates J.W., Main D.C.J. (2008). Assessment of Positive Welfare: A Review. Vet. J..

[10] Fraser D., Weary D.M., Pajor E.A., Milligan B.N. (1997). A Scientific Conception of Animal Welfare that Reflects Ethical Concerns. Anim. Welfare.

[11] Dawkins M.S. (2008). The Science of Animal Suffering. Ethology.

[12] Johnsen P.F., Johannesson T., Sandoe P. (2001). Assessment of Farm Animal Welfare at Herd Level: Many Goals, Many Methods. Acta Agr. Scand. A Anim. Sci..

[13] Main D.C.J., Kent J.P., Wemelsfelder F., Ofner E., Tuyttens F.A.M. (2002). Applications for Methods of On-Farm Welfare Assessment. Anim. Welfare.

[14] Whay H.R., Main D.C.J., Green L.E., Webster A.J.F. (2003). An Animal-Based Welfare Assessment of Group-Housed Calves on UK Dairy Farms. Anim. Welfare.

[15] Bracke M.B.M. (2007). Animal-Based Parameters are no Panacea for On-Farm Monitoring of Animal Welfare. Anim. Welfare.

[16] Rushen J., Butterworth A., Swanson J.C. (2011). Farm Animal Welfare Assurance: Science and Application. J. Anim. Sci..

[17] (2009). Welfare Quality Assessment Protocol for Cattle, Poultry and Pigs.

[18] (2011). Farm Animal Welfare Council Final Report.

[19] Vannier P., Berthe F. (2012). Editorial: The Role of EFSA in Animal Welfare. EFSA J..

[20] (2011). Labelling Food from Farm Animals: Method of Production Labels for the European Union.

[21] Bayvel A.C.D. The OIE Animal Welfare Strategic Initiative—Progress, Priorities and Prognosis. Proceedings of Global Conference on Animal Welfare: An OIE Initiative.

[22] Green T.C., Mellor D.J. (2011). Extending Ideas about Animal Welfare Assessment to Include ‘Quality of Life’ and Related Concepts. N. Z. Vet. J..

[23] Mendl M., Burman O.H.P., Paul E.S. (2010). An Integrative and Functional Framework for the Study of Animal Emotion and Mood. Proc. Roy. Soc. B Biol. Sci..

[24] Mellor D.J. (2012). Animal Emotions, Behaviour and the Promotion of Positive States. N. Z. Vet. J..

[25] Mullan S., Edwards S.A., Whay H.R., Butterworth A., Main D.C.J. (2011). A Pilot Investigation of Possible Positive System Descriptors in Finishing Pigs. Anim. Welfare.

[26] Held S.D.E., Spinka M. (2011). Animal Play and Animal Welfare. Anim. Behav..

[27] Scott E.M., Nolan A.M., Fitzpatrick J.L. (2001). Conceptual and Methodological Issues Related to Welfare Assessment: A Framework for Measurement. Acta Agr. Scand. A Anim. Sci..

[28] Mench J.A. (2008). Farm Animal Welfare in the USA: Farming Practices, Research, Education, Regulation, and Assurance Programs. Appl. Anim. Behav. Sci..

[29] Veissier I., Butterworth A., Bock B., Roe E. (2008). European Approaches to Ensure Good Animal Welfare. Appl. Anim. Behav. Sci..

[30] Blokhuis H.J., Veisser I., Miele M., Jones B. (2010). The Welfare Quality Project and Beyond: Safeguarding Farm Animal Well-Being. Acta Agr. Scand. A Anim. Sci..

[31] Main D.C.J., Mullan S., Atkinson C., Bond A., Cooper M., Fraser A., Browne W.J. (2012). Welfare Outcomes Assessment in Laying Hen Farm Assurance Schemes. Anim. Welfare.

